# A trehalase from *Zunongwangia* sp.: characterization and improving catalytic efficiency by directed evolution

**DOI:** 10.1186/s12896-016-0239-z

**Published:** 2016-01-29

**Authors:** Qipeng Cheng, Haofeng Gao, Nan Hu

**Affiliations:** College of Biotechnology and Pharmaceutical Engineering, Nanjing Tech University, Nanjing, 211800 P. R. China; State Key Laboratory of Agricultural Microbiology, College of Life Science and Technology, Huazhong Agricultural University, Wuhan, 430070 P. R. China

**Keywords:** TreZ, Marine bacterium, Error-prone PCR, Site-directed mutagenesis, Catalytic efficiency

## Abstract

**Background:**

Trehalases have potential applications in several fields, including food additives, insecticide development, and transgenic plant. In the present study, we focused on a trehalase from the marine bacterium *Zunongwangia* sp., which hydrolyzes trehalose to glucose.

**Results:**

A novel gene, *treZ* (1590 bp) encoding an α, α-trehalase of 529 amino acids was cloned from *Zunongwangia* sp., and TreZ was found to have an optimal activity at 50 °C and pH 6. The activity of TreZ was increased by the presence of NaCl, showing the highest activity (136 %) at 1 M NaCl. A variant C4 with improved catalytic activity was obtained by error-prone PCR and followed by a 96-well plate high-throughput screening. The variant C4 with two altered sites (Y227H, and R442G) displayed a 3.3 fold increase in catalytic efficiency (*k*_cat_/*K*_m_, 1143.40 mmol^−1^ s^−1^) compared with the wild type enzyme (265.91 mmol^−1^ s^−1^). In order to explore the contribution of the mutations found in variant C4 to the increased catalytic activity, two mutants Y227H and R442G were constructed by site-directed mutagenesis. The results showed that the catalytic efficiencies of Y227H and R442G were 416.78 mmol^−1^ s^−1^ and 740.97 mmol^−1^ s^−1^, respectively, indicating that both mutations contributed to the increased catalytic efficiency of variant C4. The structure modeling and substrate docking revealed that the substitution Y227H enlarged the shape of the binding pocket, to improve the binding of the substrate and the release of the products; while the substitution R442G reduced the size of the side chain and decreased the steric hindrance, which contributed to channel the substrate into the active cavity easier and promote the release of the product.

**Conclusion:**

In this study, a novel trehalase was cloned, purified, characterized, and engineered. A variant C4 with dramatically improved catalytic activity was obtained by directed evolution, and the mutation sites Y227H and R442G were found to play a significant role in the catalytic efficiency. The overall results provide useful information about the structure and function of trehalase.

**Electronic supplementary material:**

The online version of this article (doi:10.1186/s12896-016-0239-z) contains supplementary material, which is available to authorized users.

## Background

Trehalose is a non-reducing disaccharide in which the two glucose units are linked in α-α-1, 1-glycosidic linkage. It is widely distributed in nature and mainly isolated from bacteria, fungi, insects, invertebrates, and plants [1]. In living cells trehalose acts as a carbon source [2], a signal molecule [3, 4], an essential component of cell wall [5, 6]. It also protects the membranes under stress conditions [7, 8].

In the present work, *Zunongwangia* sp., a marine bacterium, which survives under high hydrostatic pressure and a low temperatures environment was explored for trehalase production [9]. In *Zunongwangia* sp. trehalose is not only a source of energy but also associated with high salt-tolerance and cold adaptation [9]. α, α-trehalases (EC 3.2.1.28) are the enzymes that specifically hydrolyze trehalose to glucose.

Based on the amino acid sequence of known and hypothetical proteins, α, α-trehalases are classified into three glycoside hydrolase (GH) families 15, 37, and 65 (http://www.cazy.org/) [10, 11]. These families belong to the clan GH-L or GH-G, but share the same classic (α/α)_6_ barrel fold, and an inverted reaction mechanism [12]. A previous study showed that over-expression of plant trehalase in Arabidopsis decreased the trehalose levels to recover from drought stress [13]. In another study, trehalose accumulation was reported to have toxic effect in *Cuscuta reflexa* [14], suggesting that the transfer of trehalase gene into plants will contribute to protect the plants under such conditions. Trehalase deficiency is a metabolic disorder in which human body is not able to convert trehalose into glucose [15]. Individuals suffering from this deficiency experience vomit, abdominal discomfort and diarrhea after consumption of trehalose rich food [16]. These reports indicated that trehalase is both a therapeutic enzyme and potential food additive.

In 2007, the three-dimensional structure of periplasmic trehalase (Tre37) from *E. coli* was explored [17]. Silva et al. first provided the evidence (site-directed mutagenesis) that trehalase from *Spodoptera frugiperda*, a member of GH family 37, has an aspartate residue (D322) and a glutamate residue (E520) as the general acid and base catalysts, respectively [18]. Error-prone-PCR and Site-directed mutagenesis are commonly used to generate a large mutant library with modified sequences and to examine the role of specific residues in enzyme activity [19, 20].

To date, to the best of our knowledge, only Silva et al. [18] have reported the mutants of trehalase from *Spodoptera frugiperda* and determined the active site. The objectives of present study were to characterize a novel Trehalase (TreZ) from *Zunongwangia* sp. and to improve its activity by directed evolution.

## Results

### Characteristics of *treZ* gene, TreZ and mutants

**Fig. 1:**
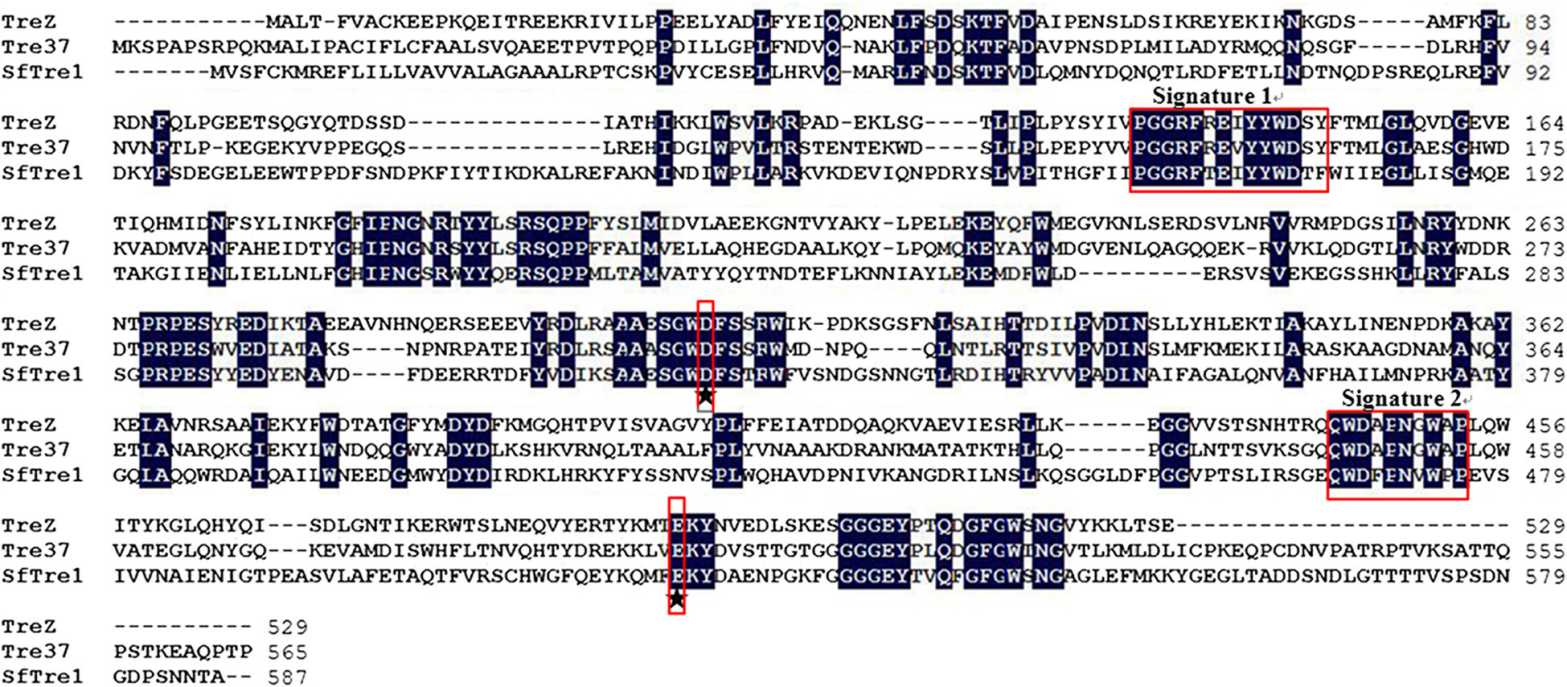
Amino acid sequence alignment of TreZ with other characterized trehalases. TreZ, TreZ investigated in this study; Tre37, trehalase from *E.coli* (EDU65093); SfTre1, trehalase from *Spodoptera frugiperda* (ABE27189). The two red rectangular boxes Signature 1 and Signature 2 represent two highly conserved sequence segments which belonging to GH37 family. Asterisks indicate the catalytic sites of Asp_306_ and Glu_494_

A novel gene *treZ* (1590 bp; GC content 36.98 %) was successfully cloned from *Zunongwangia* sp. The open reading frame encoded a protein (530 amino acids) with a predicted molecular mass of 61.3 kDa and an isoelectric point (*p*I) of 4.98. The sequence alignment indicated that TreZ showed high homology to TreZ from GH37 family and shared 62 % identity with a TreZ from *Gillisia* sp. JM1 (WP_026839117), 44 % identity with Tre37 from *E. coli* (EDU65093) and 31 % identity with SfTre1 from *Spodoptera frugiperda* (ABE27189; Fig. 1).

**Fig. 2:**
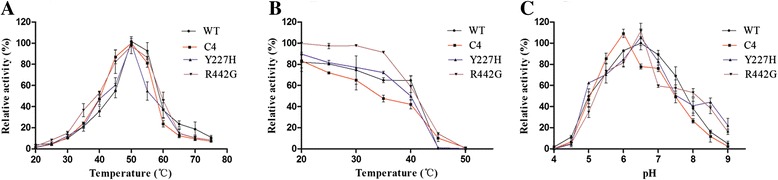
Effects of pH and temperature on enzyme activity and stability. **a** Effect of temperature on the activity of TreZ and mutants. The maximal activity was taken as 100 %. **b** Effect of temperature on the stability of TreZ and mutants. The enzyme activity without pre-treatment was taken as 100 %. **c** Effect of pH on the activity of TreZ and mutants. The maximal activity was taken as 100 %. Black line represents WT (TreZ), red line represents C4, green line represents Y227H and grey line represents R442G

The screening of mutant library was performed by a high-throughput screening. A mutant C4, exhibiting higher catalytic efficiency than the wild type, was selected from 8000 clones. Sequence analysis revealed that C4 was mutated at two sites (Y227H and R442G). To explore the effect of the single site on the catalytic activity of variant C4, two single site mutants Y227H and R442G were constructed and analyzed separately. The results showed that the purified proteins of TreZ and mutants had an identical molecular weight of ~61.31 kDa (Additional file 1: Figure S1). The optimal temperature for TreZ and mutants was 50 °C (Fig. 2a). TreZ, C4, Y227H and R442G retained more than 50 % of their original activities in the temperature range of 40 to 60 °C. Moreover, TreZ was stable after 1 h incubation below 40 °C, but it lost 90 % of its original activity at 45 and 50 °C. The mutant C4, Y227H, and R442G also showed a similar trend in thermo-stability (Fig. 2b). TreZ, C4, Y227H, and R442G showed the optimal activity at pH 6.5, 6.0, 6.5 and 6.5 respectively (Fig. 2c). TreZ and mutants retained more than 60 % of the original activity in a pH range of 5.0–8.0.

### The substrate specificity and kinetic parameters of TreZ and mutants

A study of specific activity with different substrates showed that TreZ was only highly specific to trehalose (257.6 U/mg) and was non catalytic towards other substrates (Table 1).

**Table 1:** The substrate specificity of TreZ

Substrate	Specific activity (U/mg)
Trehalose	257.6
Sucrose	0
Maltose	0
Lactose	0
Cellobiose	0

Kinetic parameters of TreZ and mutants were studied under optimal conditions (Table 2). Mutant C4 showed a 61 % decrease in *K*_m_, a 65 % increase in *k*_*cat*_, and a 3.3-fold increase in *k*_*cat*_/*K*_m_. Mutant Y227H showed a 27 % decrease in *K*_m_, a 14 % increase in *k*_*cat*_, and a 0.57 fold increase in *k*_*cat*_/*K*_m_. Mutant R442G showed a 56 % decrease in *K*_m_, a 22 % increase in *k*_*cat*_, and a 1.78 fold increase in *k*_*cat*_/*K*_m_. The catalytic efficiencies of mutant C4, Y227H and R442G were 1143.40 mmol^−1^ s^−1^, 416.78 mmol^−1^ s^−1^ and 740.97 mmol^−1^ s^−1^, respectively. The total sum of the catalytic efficiencies of Y227H and R442G was nearly equal to the catalytic efficiency of mutant C4. It indicated that the two sites (Y227H, R442G) together contributed to the increased catalytic efficiency of mutant C4.

**Table 2:** Steady-state kinetic parameters for wild-type TreZ and mutant

Enzyme	*K*_m_ (mmol l^−1^)	*k*_*ca*t_ (s^−1^)	*k*_*cat*_/*K*_m_ (mmol^−1^ s^−1^)
Wild-type(TreZ)	0.99 ± 0.04	263.25 ± 0.91	265.91 ± 1.21
C4	0.3793 ± 0.03	433.69 ± 0.82	1143.40 ± 1.67
Y227H	0.7194 ± 0.05	299.83 ± 1.39	416.78 ± 1.57
R442G	0.4317 ± 0.03	319.88 ± 0.95	740.97 ± 1.64

The data are the average of three replicates

### Effects of metal ions and chemical reagents on TreZ

The effects of metal ions and chemical regents on TreZ are shown in Table 3, indicating that the enzyme activity was slightly inhibited by Co^2+,^ EDTA and ATP (5,10 mM), and strongly inhibited by Fe^3+^ (5,10 mM), Cu^2+^, Zn^2+^ (1, 5, 10 mM), and ADP (5,10 mM). In contrast, Ni^+^ strongly enhanced the activity of TreZ at 1 mM (158.9 ± 0.69 %), moderately at 5 mM (125.9 ± 1.07 %), and weakly at 10 mM (112.7 ± 8.1 %). K^+^, Mg^2+^, Ca^2+^, and Ba^2+^ increased TreZ activity to 127.4 ± 6.69 %, 116.1 ± 5.3 %, 134.4 ± 4.2 % and 135.5 ± 3.6 %, respectively.

**Table 3:** Effect of metal ions and chemical reagents on TreZ^a^

The reagent	Concentration/Relative activity (%)		
	1 mM	5 mM	10 mM
K^+^	106.64 ± 1.58^c^	127.44 ± 6.69	94.71 ± 1.58
Ni^+^	158.92 ± 0.69	125.97 ± 1.07	112.76 ± 8.14
Mg^2+^	92.46 ± 2.38	100.85 ± 1.02	116.15 ± 5.36
Ca^2+^	105.27 ± 5.69	134.43 ± 4.20	126.31 ± 7.24
Fe^3+^	65.67 ± 0.12	7.17 ± 0.46	4.91 ± 2.15
Co^2+^	94.55 ± 3.60	42.23 ± 0.08	38.46 ± 3.30
Cu^2+^	47.47 ± 4.58	3.91 ± 2.47	-^b^
Zn^2+^	19.96 ± 2.27	18.86 ± 0.50	14.3 ± 3.44
Ba^2+^	105.25 ± 2.67	135.54 ± 3.59	111.94 ± 0.63
EDTA	70.48 ± 5.36	78.75 ± 9.23	44.45 ± 5.26
ATP	155.65 ± 4.78	76.71 ± 6.00	55.75 ± 8.77
ADP	139.02 ± 0.82	9.09 ± 2.02	-^b^

^a^:All assays were preformed in the standard conditions and the activity without additional reagent and ions was taken as 100 %; ^b^:unmeasured data; ^c^:Relative activity ± the standard deviation

**Fig. 3:**
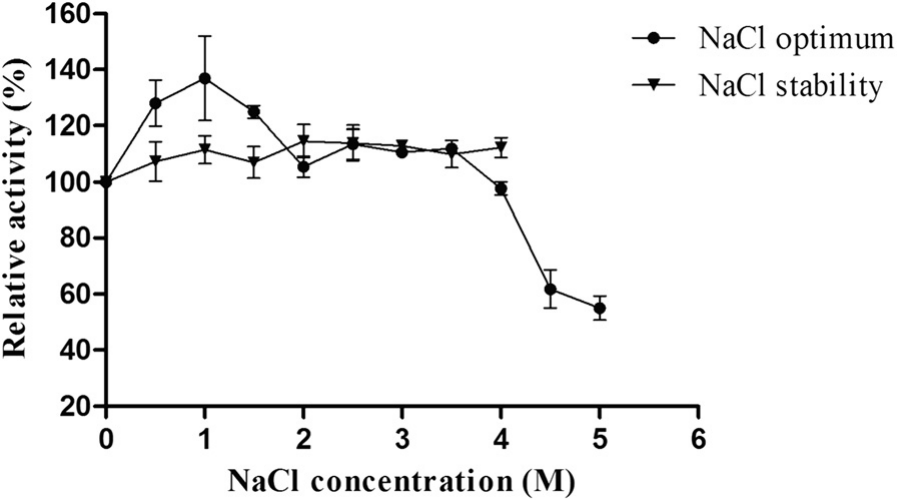
Effects of NaCl on enzyme activity (●) and stability (▼). Enzyme activity was measured in 50 mM Tris–HCl buffer (pH7.0) containing different concentrations of NaCl (0–5 M) at 50 °C, with reaction mixture containing no NaCl as 100 %. The stability assay was conducted after 24 h incubation of enzyme in 0–4 M NaCl. The activity without incubation was taken as control

The activity of TreZ increased significantly to 136 % at 1 M NaCl, and more than 100 % of its original activity was retained at 0.5–3.5 M NaCl, and 55 % of its original activity was maintained at 5 M NaCl (Fig. 3). Furthermore, TreZ was very stable under high salt conditions, and showed no considerable loss in activity even after 24 h incubation in 0.5–4 M NaCl.

### Substrate docking analysis of TreZ and mutants

**Fig. 4:**
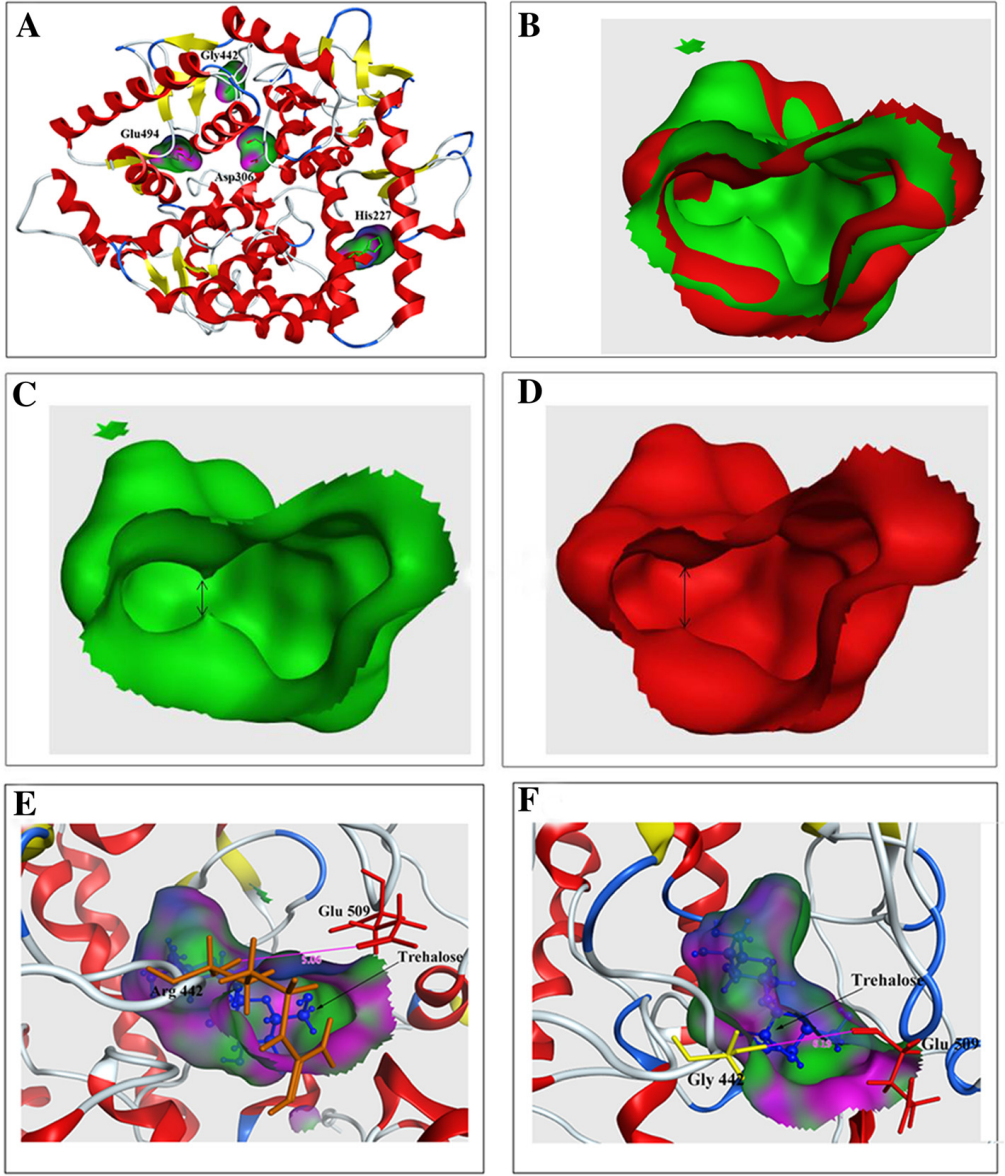
**a** Location of amino acid substitutions in the predicted modules. The catalytic sites (Asp_306_, Glu_494_) and substitutions are in mutant C4. **b** The binding pocket superimposition of wild type (green color) and Y227H (red color). **c** The binding pocket of wild type (green color). **d** The binding pocket of Y227H (red color). **e** Docking analysis of TreZ-trehalose complex showing the position of trehalose in active cavity and the location of residue Arg_442_. **f** Docking analysis of R442G-trehalose complex showing the position of trehalose in active cavity and the location of residue Gly_442_

The models of TreZ and mutant enzymes were constructed based on the structure of trehalase from *E. coli* (Tre37; PDB code: 2WYN) with a 45.27 % sequence identity [21]. As expected, the structure of the wild-type enzyme with a classical (α/α) _6_ barrel fold and two catalytic residues (Asp306 and Glu494) were located in the inner surfaces of the central cavity (Fig. 4a). To identify the possible molecular basis for the enhancement of catalytic efficiency, we constructed a docking model of the Y227H and R442G-trehalose complex based on the homology model (Fig. 4b, c, d, e and f). The substrate docking analysis indicated that the residue 227 was located in α helix domain belonging to an (α/α) _6_ barrel, and another residue 442 was located on the loop between two β-sheets. In the substitution Y227H, the replacement of tyrosine by histidine obviously enlarged the shape of the binding pocket (Fig. 4b, c and d). Interestingly, when glycine was replaced by arginine at site 442G, the nearest distance between residue 442 and residue 509 (another residue located over the active cavity, which is on the opposite side of residue 442, see Fig. 4e, f) increased from 5.06 to 6.19 Å.

## Discussion

In the present study, TreZ from *Zunongwangia* sp. was isolated and expressed in *E. coli* BL21 (DE3). The optimum temperature for TreZ is 50 °C, which is higher than that of many previously reported trehalases, such as those in *Apis mellifera* L 7 °C [22], *Rhodotorula rubra* 30 °C [23], *Saccharomyces cerevisiae* 40 °C [24], and *Rhizopus microsporus var. rhizopodiformis* 45 °C [25]. However, a trehalase from a thermophilic bacterium *Rhodothermus marinus* has a higher optimal temperature of 88 °C than TreZ [26].

Another remarkable characteristic of TreZ is its extreme salt-tolerance (Fig. 3). The enzyme was found to be active over a wide NaCl concentration range (0–5 M exhibiting the maximum activity at 1 M NaCl (136 %) and retaining more than 100 % of its original activity at 0.5–3.5 M NaCl. This behavior is similar to that of a xylanase and two amylases from *Zunongwangia* sp., which showed the optimum activity at 3, 1.5 and 2 M NaCl, respectively [27–29]. TreZ retains activity with or without NaCl and thus differs from other halophilic enzymes which require salt to remain active and stable, indicating that TreZ is a salt-tolerant enzyme. To date, several reports have been published about the trehalases, whose activity was enhanced by Na^+^ [30, 31]. Furthermore, TreZ is relatively stable after 24 h incubation in high salinity conditions (Fig. 3). A similar salt tolerance behavior was also observed in xylanase and amylases from *Zunongwangia* sp. [27–29]. Additionally, TreZ activity was also enhanced by K^+^, Ni^+^, Mg^2+^, Ca^2+^, and Ba^2+^ and strongly inhibited by Fe^3+^, Cu^2+^, Zn^2+^ and ADP (10 mM).

In this study, we improved the catalytic efficiency of TreZ (1143.4 mmol^−1^ s^−1^) by directed evolution. When ompared to previously reported trehalases in terms of the catalytic efficiency of mutant C4 is higher than the trehalases (262–730 mmol^−1^ s^−1^) from *Metarhizium strains* [32] and Tre37 (485 mmol^−1^ s^−1^) from *E.coli* [21], but lower than the trehalase (1273 mmol^−1^ s^−1^) from *Apis mellifera* L [22] and trehalase (2400 mmol^−1^ s^−1^) from *Spodoptera frugiperda* [18].

Despite many studies trehalases, only a few further engineered trehalases have been reported. Silva et al. reported that three Arg residues (R169, R222 and R287) are essential for trehalase activity from *Spodoptera frugiperda* [18]*.* In this study, a mutant C4 (Y227H and R442G) dramatically improved catalytic activity by directed evolution. To understand the relationship between single mutant site and catalytic efficiency, the single site mutants were constructed separately, respectively. The results showed that Y227H and R442G played an important role on the catalytic efficiency of trehalase.

The structure modeling analysis and substrate docking (Fig. 4a, b, c and d) of Y227H revealed a subtle modification of the shape of the binding pocket. According to Morley et al. [33], the replacement like Y227H far away from the active site may remodel the site arrangement and lead to fine alterations in the protein backbone and side chain, which altered the secondary structure of protein, and also produced a subtle change in the shape of the binding pocket, finally leading to dramatic changes in the catalytic activity of enzyme. The modeled TreZ and mutant structures (Fig. 4a) and the docking analysis of the substrate (Fig. 4e, f) showed that the residue 442 is in the vicinity of the entrance to the active site. In R442G, the replacement of arginine by glycine reduced the size of the side chain of residue 442. Due to the reduction of the side-chain functional groups, the nearest distance between residue 442 and residue 509 become longer, which decreased the steric hindrance, contributed to channel the substrate into the central binding pocket easier and promoted the release of product, finally leading to the improvement of the catalytic efficiency of trehalase.

## Conclusion

In this study, a novel salt-tolerant trehalase from *Zunongwangia* sp. was cloned, purified, characterized, and engineered. Moreover, we found that the mutation sites Y227H and R442G make synergic contributions to the catalytic activity of mutant C4 and explored that the single mutant site affects the catalytic activity. These results provided useful some insight into the relationship between structure and function of the trehalase.

## Methods

### Bacterial strains, plasmids and medium

D-trehalose and D-glucose were purchased from Sigma-Aldrich (St. Louis, MO, USA); Restriction endonucleases from Takara (Japan); T4 DNA ligase and pfu DNA polymerase from Transgene (Beijing, China); and DNA purification Kits from Axygen (USA). The PreScission protease and the GST-binding Resin were procured from GE Healthcare (USA) and Merck (Germany), respectively. GST-Bind Purification kit was purchased from Novagen (Germany).

*Zunongwangia* sp. was isolated from seawater. *E. coli* strains DH5α and BL21 (DE3) were used as gene cloning and protein expression hosts, respectively. The plasmid vector for cloning and expression was pGEX-6P-1 (GE Healthcare, USA). *Zunongwangia* sp. was grown in Luria-Bertani (LB) medium containing 20 g l^−1^ NaCl at 26 °C. The strains of *E. coli* were grown on LB medium or LB agar plates at 37 °C with Ampicillin (100 μg ml^−1^).

### Cloning of *treZ* gene

The genomic DNA of *Zunongwangia* sp. was purified and used as a template for amplification of TreZ gene (*treZ*). The primers were designed on the basis of putative TreZ gene from *Zunongwangia profunda* SM-A87 (GenBank CP001650) which was sequenced by Qin et al. [9]. The pair of primers (TreZ-F, TreZ-R) was listed in Additional file 1: Table S1. The amplification was performed by following the PCR(s) program: (i) 94 °C for 4 min, (ii) 30 cycles of 94 °C for 30 s, 49 °C for 30 s, and 72 °C for 96 s, and (iii) 72 °C for 10 min. The amplified products were purified and digested with *Eco*R I/*Xho* I and ligated into the same restriction site of pGEX-6p-1 to generate recombinant plasmid pGEX-6p-*treZ*. The pGEX-6P-*treZ* was transformed into *E. coli* BL21 (DE3) for protein expression and purification. The nucleotide and protein sequences were analyzed using the BLAST tool in the NCBI website. Multiple sequence alignment was performed using the DNAMAN software package.

### Construction of mutant library

Error-prone PCR was used to construct the randomly mutant *treZ* gene library. The PCR mixture (100 μl) was composed of *Taq* buffer containing 5 mM MgCl_2_, 20 ng template pGEX-6p-*treZ*, 1 mM dNTPs, 0.2 mM dTTP and dCTP, 0.2 mM MnCl_2_, 2.5 units of Taq DNA polymerase, and 0.4 μM primers (TreZ-F and TreZ-R). The PCR reaction was carried out at an initial temperature of 94 °C for 4 min, followed by 30 cycles of (94 °C for 30 s, 46 °C for 30 s, and 72 °C for 96 s) and a final elongation at 72 °C for 10 min. The amplified PCR products were digested by *Eco*R I and *Xho* I and cloned into plasmid pGEX-6p-1, which were transformed into *E. coli* DH5α to obtain the mutant library.

### Screening of library

Transformants of the TreZ mutant library were spread on LB plates containing 100 μg ml^−1^ ampicillin and incubated for 14 h at 37 °C. The colonies were picked up with sterile toothpick(s) and resuspended separately in a 96-deep-well plate containing 0.6 mL liquid LB medium and 100 μg ml^−1^ ampicillin. After incubation for 20 h at 37 °C, 180 μl of fresh LB medium (0.1 mM IPTG and T7 phage) was added to each well [34, 35], followed by incubation for 6 h at 28 °C under shaking at 180 r.p.m. Subsequently, each cell suspension from the 96-deep well (150 μl) was transferred to another 96-deep-well flat-bottom block with each well containing 250 μl of trehalose (20 mM). After incubation for 10 min at 50 °C, reaction was stopped by adding 200 μl of DNS. Finally, the mixture was boiled for 5 min, and absorbance was determined at A_540_ using a Multiskan Spectrum spectrophotometer (Thermo Scientific, Vantaa, Finland). The clones with a higher absorbance value than that of the wild-type enzyme were selected for further evaluation.

### Site-directed mutagenesis

The site-directed mutagenesis was carried out by one-step overlap PCR, using the plasmid pGEX-6p-*treZ* as a template and primers designed from pairs of complementary oligonucleotides containing desired mutants (Additional file 1: Table S1). The PCR program was set as follows: denaturation at 97 °C for 2 min, 20 cycles of 20 s at 95 °C, 30 s at 54 °C, 1 min 40 s at 72 °C, followed by a 7-min extension at 72 °C and 10-min preservation at 15 °C [36]. After PCR reaction, the amplified products were mixed with *Dpn* I to digest the wild-type templates, followed by incubation for 12 h at 37 °C, and then transformed into *E. coli* DH5α cells. The selected transformants were sequenced, and transformed into *E. coli* BL21 (DE3) for enzyme expression and purification.

### Protein expression and purification

The recombinant plasmid pGEX-6P-*treZ* and mutants were expressed in *E. coli* BL21 (DE3) cells. A single colony grown on an LB plate (containing 100 μg ml^−1^ ampicillin) was used to prepare the seed culture, 20 ml of which was transferred to 1 L of LB medium (containing 100 μg ml^−1^ ampicillin) and grown at 37 °C until the absorbance reached 0.6 at 600 nm. Then gene expression was induced by adding IPTG (0.1 mM) into the culture, and further incubated at 18 °C for 16 h. Cells were harvested by centrifugation and resuspended in cold phosphate-buffered saline (PBS, 140 mM NaCl, 2.7 mM KCl, 10 mM Na_2_HPO_4_, and 1.8 mM KH_2_PO_4,_ pH 7.0) followed by cell disruption using a French pressure cell technique. The cell debris was spun down at 12,000 × g for 30 min at 4 °C, followed by purifying the glutathione-S-transferase (GST) tagged TreZ protein in the supernatant, and adding the 3C protease (PreScission, Pharmacia) to remove the GST tag using GST fusion protein purification kit according to manufacturer’s instruction. Finally, the purified protein was eluted in 1 ml of PBS (pH 7.0). All purification steps were carried out at 4 °C. The homogeneity of purification and the molecular mass of the enzyme were determined by 12 % SDS-PAGE (sodium dodecyl sulfate-polyacrylamide gel electrophoresis) and the protein concentration was measured by the Bradford method [37] using bovine serum albumin (BSA) as standard.

### Enzymatic assay and kinetic parameters assay

The activity of TreZ was determined by a modified method reported by Pereira et al. [38]. The amount of reducing D-glucose was determined by the 3,5-dinitrosalicylic acid (DNS) method and GOD-POD method [39, 40]. One unit (U) of enzyme activity was defined as the amount of enzyme required to produce 1 μmol of glucose in one minute under standard assay conditions. The specific activity was expressed as units per mg protein (U/mg).

The substrate specificity was tested by using the substrates of trehalose, sucrose, maltose, lactose and cellobiose. The enzyme assay was carried out under standard assay conditions.

The *K*_m_ and *k*_*cat*_ values of TreZ and the mutants were determined by measuring the enzyme activity using trehalose ranging from 0.2 to 4.0 mM L^−1^ under optimal reaction conditions. The data were estimated by the Lineweaver-Burk plot method using Graph-pad Prism software.

### Effects of temperature, pH, metal ions, and chemical reagents on trehalase activity

The effect of temperature on enzyme activity was assayed at a temperature range from 20 to 75 °C. To determine the thermostability, the enzyme was incubated for 1 h at the indicated temperatures, and residual activities were measured under standard assay conditions. The effect of pH on the purified TreZ was evaluated under TreZ activity assay conditions at the optimal temperature in a pH range of 4.0–9.0, using two different buffers:0.2 M Na_2_HPO_4_/0.1 M citric acid buffer for pH 4.0–8.0, and 0.05 M Na_2_B_4_O_7_ · 10H_2_O/0.2 M NaOH buffer for pH 8.0–9.0.

The effect of salt concentration on enzyme activity was tested in a Tris–HCl buffer (50 mM; pH 7.0) containing different concentrations of NaCl (0–5 M) at 50 °C. The influence of NaCl on enzyme stability was studied by measuring the residual activity after pre-incubation of enzyme solution with 0–4 M NaCl at 20 °C for 24 h. Untreated enzyme was considered as control. The effects of different metal ions and chemical reagents on enzyme activity were evaluated under optimal conditions at 1, 5 and 10 mM concentration; the enzyme without any reagent treatment was taken as control.

### Homology model

The three dimensional (3D) structures of TreZ and mutants were predicted by SWISS-MODEL (http://swissmodel.expasy.org/) [41] and modeled based on the reported crystal structure of Trehalase Tre37 from *E. coli* (PDB code: 2WYN). Docking analyses of substrate with TreZ and mutants were performed by Molecular Operating Environment (MOE) 2009 (Chemical Computing Group Inc., Montreal, Canada).

## Additional file

Additional file 1: Figure S1. SDS-PAGE analysis of the purified proteins: TreZ, C4, Y227H and R442G; **Table S1.** Primers used for plasmid construction and the site-directed mutagenesis: *tre*Z-F, *tre*-R; Y227H-F, Y227-R; R442G-F, R442G-R. (DOC 638 kb)

## References

[1] Elbein AD (1974). The metabolism of α, α-trehalose. Adv Carbohydr Chem Biochem.

[2] Dawes EA, Senior PJ (1973). The role and regulation of energy reserve polymers in micro-organisms. Adv Microbial Physiol.

[3] Paul MJ, Primavesi LF, Jhurreea D, Zhang Y (2008). Trehalose metabolism and signaling. Annu Rev Plant Biol.

[4] Gancedo C, Flores CL (2004). The importance of a functional trehalose biosynthetic pathway for the life of yeasts and fungi. FEMS Yeast Res.

[5] Brennan PJ, Nikaido H (1995). The envelope of mycobacteria. Annu Rev Biochem.

[6] Shimakata T, Minatogawa Y (2000). Essential role of trehalose in the synthesis and subsequent metabolism of corynomycolic acid in *Corynebacterium matruchotii*. Arch Biochem Biophys.

[7] Argüelles JC (2000). Physiological roles of trehalose in bacteria and yeasts: a comparative analysis. Arch Microbiol.

[8] Crowe JH, Crowe LM, Chapman D (1984). Preservation of membranes in anhydrobiotic organisms: the role of trehalose. Science.

[9] Qin Q-L, Zhang X-Y, Wang X-M, Liu G-M, Chen X-L, Xie B-B (2010). The complete genome of *Zunongwangia profunda* SM-A87 reveals its adaptation to the deep-sea environment and ecological role in sedimentary organic nitrogen degradation. BMC Genomics.

[10] Henrissat B, Bairoch A (1993). New families in the classification of glycosyl hydrolases based on amino acid sequence similarities. Biochem J.

[11] Cantarel BL, Coutinho PM, Rancurel C, Bernard T, Lombard V, Henrissat B (2009). The Carbohydrate-Active EnZymes database (CAZy): an expert resource for glycogenomics. Nucleic Acids Res.

[12] Kötzler MP, Hancock SM, Withers SG. Glycosidases: Functions, Families and Folds. eLS. 2014. In press

[13] Van Houtte H, Vandesteene L, López-Galvis L, Lemmens L, Kissel E, Carpentier S (2013). Overexpression of the trehalase gene AtTRE1 leads to increased drought stress tolerance in *Arabidopsis* and is involved in abscisic acid-induced stomatal closure. Plant Physiol.

[14] Veluthambi K, Mahadevan S, Maheshwari R (1981). Trehalose toxicity in *Cuscuta reflexa* Correlation with low trehalase activity. Plant Physiol.

[15] Murray IA, Coupland K, Smith JA, Ansell ID, Long RG (2000). Intestinal trehalase activity in a UK population: establishing a normal range and the effect of disease. British J Nutr.

[16] Kleiman R, Goulet O, Mieli-Vergani G, Sanderson I, Sherman P, Shneider B (2008). Walker’s pediatric gastrointestinal disease: physiology, diagnosis, management. Hamilton: BC Decker INC.

[17] Gibson RP, Gloster TM, Roberts S, Warren RAJ, Storch de Gracia I, Garcia A (2007). Molecular basis for trehalase inhibition revealed by the structure of trehalase in complex with potent inhibitors. Angew Chem Int Edit.

[18] Silva MC, Terra WR, Ferreira C (2010). The catalytic and other residues essential for the activity of the midgut trehalase from *Spodoptera frugiperda*. Insect Biochem Mol Biol.

[19] Wang J, Zhang Q, Huang Z, Liu Z (2013). Directed evolution of a family 26 glycoside hydrolase: endo-beta-1, 4-mannanase from *Pantoea agglomerans* A021. J Biotechnol.

[20] Liu H, Naismith JH (2008). An efficient one-step site-directed deletion, insertion, single and multiple-site plasmid mutagenesis protocol. BMC Biotechnol.

[21] Cardona F, Goti A, Parmeggiani C, Parenti P, Forcella M, Fusi P (2010). Casuarine-6-O-α-d-glucoside and its analogues are tight binding inhibitors of insect and bacterial trehalases. Chem Commun.

[22] Lee J-H, Saito S, Mori H, Nishimoto M, Okuyama M, Kim D (2007). Molecular cloning of cDNA for trehalase from the European honeybee, *Apis mellifera* L., and its heterologous expression in *Pichia pastoris*. Biosci Biotechnol Biochem.

[23] Mansure JJ, Silva JT, Panek AD (1992). Characterization of trehalase in *Rhodotorula rubra*. Biochem In.

[24] Biswas N, Ghosh AK (1996). Characterisation of an acid trehalase of *Saccharomyces cerevisiae* present in trehalase-sucrase aggregate. BBA-Gen Subjects.

[25] de Aquino ACMM, Peixoto-Nogueira SC, Jorge JA, Terenzi HF, de Moraes MLT (2005). Characterisation of an acid trehalase produced by the thermotolerant fungus *Rhizopus microsporus* var. rhizopodiformis: Biochemical properties and immunochemical localisation. FEMS Microbiol Lett.

[26] Jorge CD, Sampaio MM, Hreggvidsson GÓ, Kristjánson JK, Santos H (2007). A highly thermostable trehalase from the thermophilic bacterium *Rhodothermus marinus*. Extremophiles.

[27] Liu X, Huang Z, Zhang X, Shao Z, Liu Z (2014). Cloning, expression and characterization of a novel cold-active and halophilic xylanase from *Zunongwangia profunda*. Extremophiles.

[28] Qin Y, Huang Z, Liu Z (2014). A novel cold-active and salt-tolerant α-amylase from marine bacterium *Zunongwangia profunda*: molecular cloning, heterologous expression and biochemical characterization. Extremophiles.

[29] Wu G, Qin Y, Cheng Q, Liu Z (2014). Characterization of a novel alkali-stable and salt-tolerant α-amylase from marine bacterium *Zunongwangia profunda*. J Mol Catal B-Enzym.

[30] García NAT, Iribarne C, López M, Herrera-Cervera JA, Lluch C (2005). Physiological implications of trehalase from *Phaseolus vulgaris* root nodules: partial purification and characterization. Plant Physiol Biochem.

[31] Dmitryjuk M, Żółtowska K (2003). Purification and characterization of acid trehalase from muscle of *Ascaris suum* (Nematoda). Comp Biochem Physiol B Biochem Mol Biol.

[32] Cln S, Donaldson NBidochka MJ (2004). Nucleotide sequence variation does not relate to differences in kinetic properties of neutral trehalase from the insect pathogenic fungus *Metarhizium anisophae*. Curr Microbiol.

[33] Morley KL, Kazlauskas RJ (2005). Improving enzyme properties: when are closer mutations better?. Trends Biotechnol.

[34] Wang Y, Feng S, Zhan T, Huang Z, Wu G, Liu Z (2013). Improving catalytic efficiency of endo-β-1, 4-xylanase from *Geobacillus stearothermophilus* by directed evolution and H179 saturation mutagenesis. J Biotechnol.

[35] Tarahovsky Y, Ivanitsky G, Khusainov A (1994). Lysis of *Escherichia coli* cells induced by bacteriophage T4. FEMS Microbiol Lett.

[36] Xu H, Qin Y, Huang Z, Liu Z (2014). Characterization and site-directed mutagenesis of an alpha-galactosidase from the deep-sea bacterium *Bacillus megaterium*. Enzyme Microb Technol.

[37] Bradford MM (1976). A rapid and sensitive method for the quantitation of microgram quantities of protein utilizing the principle of protein-dye binding. Anal Biochem.

[38] Pereira MG, Guimaraes LH, Furriel RP, Polizeli Mde L, Terenzi HF, Jorge JA (2011). Biochemical properties of an extracellular trehalase from *Malbranchea pulchella* var. Sulfurea. J Microbiol.

[39] Miller GL (1959). Use of dinitrosalicylic acid reagent for determination of reducing sugar. Anal Biochem.

[40] Bergmeyer H, Bernt E (1974). Determination of glucose with glucose oxidase and peroxidase. Methods of Enzym Anal.

[41] Biasini M, Bienert S, Waterhouse A, Arnold K, Studer G, Schmidt T (2014). SWISS-MODEL: modelling protein tertiary and quaternary structure using evolutionary information. Nucleic Acids Res.

