# Supplementary material for: A trehalase from Zunongwangia sp.: characterization and improving catalytic efficiency by directed evolution
Source: BMC Biotechnol. 2016 Jan 29;16:9. doi: 10.1186/s12896-016-0239-z (PMC4731906; doi:10.1186/s12896-016-0239-z)
Supplement: Additional file 1: Figure S1. — SDS-PAGE analysis of the purified proteins: TreZ, C4, Y227H and R442G; Table S1. Primers used for plasmid construction and the site-directed mutagenesis: treZ-F, tre-R; Y227H-F, Y227-R; R442G-F, R442G-R. (DOC 638 kb) [file 12896_2016_239_MOESM1_ESM.doc]

Additional file 1

Fig. S1


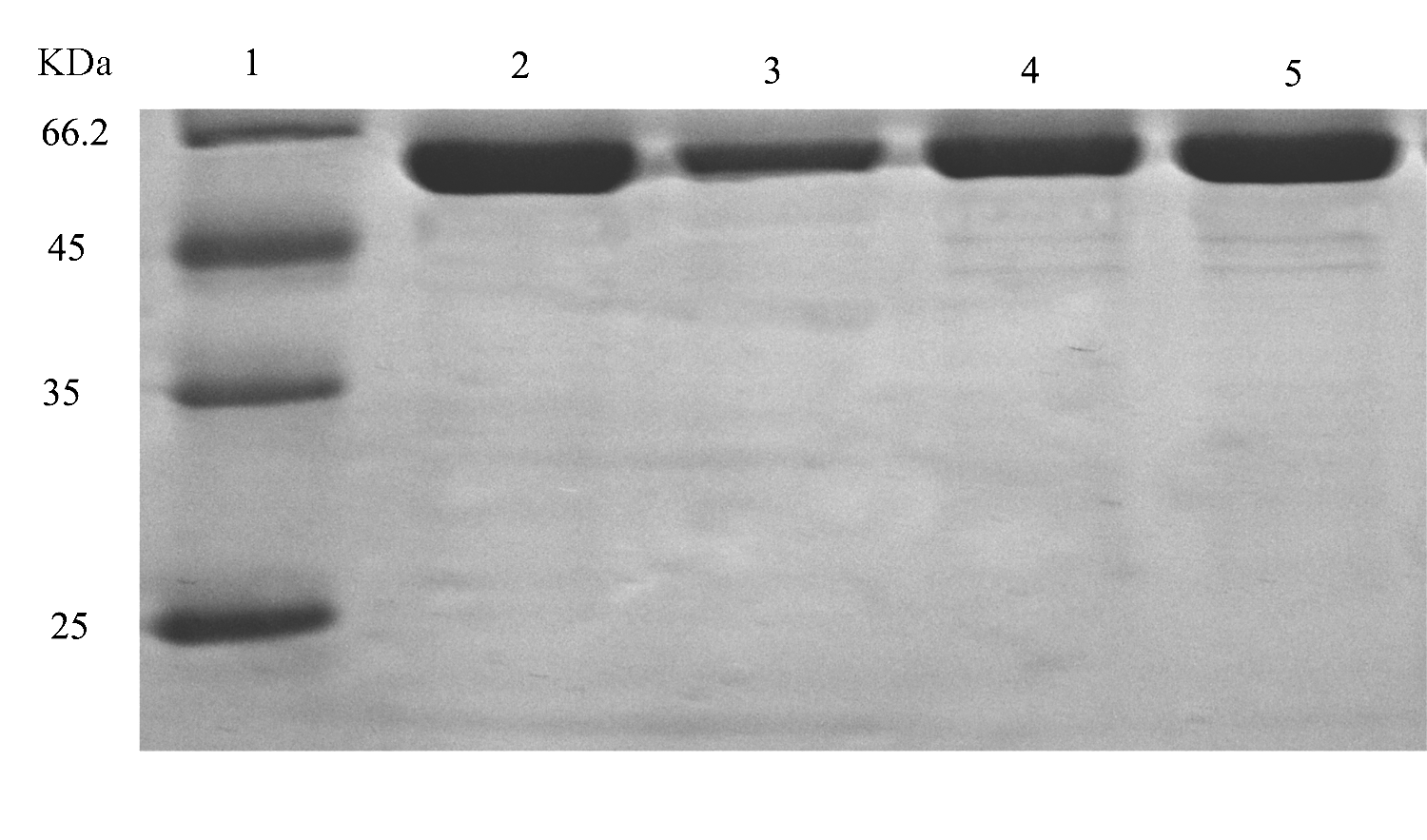


**Legends to figure**

**Fig. 1:** SDS-PAGE analysis of the purified proteins. Lane 1: the standard protein maker; Lane 2: TreZ; Lane 3: C4; Lane 4: Y227H; Lane 5: R442G.

**Table S1**

Primers used for plasmid construction and the site-directed mutagenesis

| Target | Sequence(5’-3’) |
| --- | --- |
| *treZ-F* | CCGGAATTC ATGGCTTTAACTTTTGTAGCT |
| *treZ-R* | CCGCTCGAG TTATTCAGACGTTAATTTCTT |
| Y227H-F | GAATTAGAAAAAGAACATCAATTTTG |
| Y227H-R | GTTCTTTTTCTAATTCTGGTAAGTAT |
| R442G-F | ACTTCCAATCATACCGGGCAGCAATG |
| R442G-R | CGGTATGATTGGAAGTACTCACTACT |

Note. The *Eco*R I/*Xho* I restriction site and the mutated sites are underlined.
